# Behavior change communication model enhancing parental practices for improved early childhood growth and development outcomes in rural Armenia – A quasi-experimental study

**DOI:** 10.1016/j.pmedr.2019.100820

**Published:** 2019-02-10

**Authors:** Alfonso Rosales, Viktoria Sargsyan, Karine Abelyan, Arax Hovhannesyan, Kristine Ter-Abrahanyan, Kimberly Quanstrum Jillson, Dennis Cherian

**Keywords:** Behavior change, Parental practices, Early childhood development, Quasi-experimental study

## Abstract

The latest evidence demonstrates the importance of nurturing care from conception to lay a strong foundation for children's cognitive, socio-emotional and physical well-being. The interventions enhancing parental practices in children's health and growth, protection from neglect, abuse, and injury have lifelong impact on health, learning, economic productiveness outcomes. Existing maternal and child health delivery platforms might potentially be utilized to integrate Early Childhood Development interventions. However, there is a dearth of studies demonstrating the feasibility and effectiveness of an integrated MCH and ECD model. ECD component was integrated into MCH program activities, implemented and tested in Armenia. For 14 months, all mothers of children aged 0 to 23 months (1300) living in 43 communities in Gegharkunik province (Armenia) participated in the study. Twenty-three intervention communities (680 children) received added ECD package to MCH intervention, and 20 control communities (630 children) received only MCH intervention. We used a quasi-experimental intervention-control design, with pre-and post-data collected. Variables measured and compared were related to child development, nutrition status, parental child care (stimulation, discipline) and nutrition practices.

Intervention sites showed 83% higher odd of total ECD composite score (cognitive, language, motor) compared to children in the control sites. Child caregivers had better child care, nutrition practices and early learning support than controls. No change was found in discipline practices and stunting rates. MCH-ECD integrated model is an effective delivery platform for improving parenting behavior, child growth, and development.

## Highlights

• Children exposed to intervention had better composite development outcomes.
• Effects larger for children living in socio-economic depressed geographical areas.
• The parenting component on nurturing care improved nutrition-stimulation practices.
• No significant impact on chronic malnutrition prevalence among children under 2.
• No significant impact on safety-related component (violating discipline).

## 1 Introduction

More than 200 million children under five in the developing world do not fulfill their potential for development (Grantham-McGregor et al., 2007), resulting in a total loss of $196 billion in annual productivity (Engle et al., 2011). Because of poverty, malnutrition, and learning environments that do not provide enough responsive stimulation, children develop poorly or fail to develop critical thinking and learning skills. The absence of these skills adversely affects their learning, school performance outcomes, and their success in life (UNICEF, 2006). The early age groups (0–5 years) are predominantly covered by the health sector, mainly for survival and growth, but health care providers often fail to detect problems with cognitive, language, and socioemotional development. In contrast, if child development is closely monitored in the early years, children not only perform well in school but also contribute, when adults, to their nation's economic productivity (Campbell et al., 2014).

The latest evidence demonstrates the importance of nurturing care from conception to lay a strong foundation for children's cognitive, socioemotional, and physical well-being (Walker et al., 2011). Children in low- and middle-income countries such as Armenia are at greater risk of sub-optimal development (WHO, 2018). Many children in Armenia do not reach their full development potential because of poor parenting behaviors and insufficient brain stimulation. Simple family interactions like playing, singing, and reading with young children are not perceived as important aspects of child care in typical Armenian culture. Despite the dense network of healthcare facilities and an extensively available medical workforce nationwide, Early Childhood Development (ECD) services are poorly implemented. The ECD screening tool “Ireton,” used to measure and detect 0–5 child development progress and deviations, is often not used or is inappropriately used by the primary health care providers (PHCPs). The PHCPs rarely counsel families or promote ECD. This is exacerbated by an almost 20% stunting rate (Armenia Demographic and Health Survey, 2010), and around 50% rate of anemia (American University of Armenia, 2013) among children under five.

While very few integrated ECD programs have been rigorously evaluated in Armenia, there is evidence that such programs can produce lasting, effective change. The American and Armenian Red Cross Societies implemented an integrated child illness prevention program, training peer educators and counseling approximately 5000 caretakers of children under five on key nutrition and health practices. A 2009 study of this project found an increase of 31% in exclusive breastfeeding, and of 30% in maternal knowledge of the signs of childhood illness (Thompson and Harutyunyan, 2009). Existing maternal and child health delivery platforms might potentially be used to integrate ECD interventions. However, there is a dearth of studies demonstrating the feasibility and effectiveness of integrated traditional delivery platforms and ECD intervention and its related impact on behavioral change.

In November 2014, World Vision Armenia received a grant to implement research assessing the effectiveness of the integration of an ECD counseling model within an integrated maternal, neonatal, and child strategy intervention in Armenian rural communities. Specifically, a social behavioral change (SBC) delivery platform model (focused solely on nutrition and stunting reduction, later referred to as “7–11” model) was compared to a combined SBC platform model (adding ECD intervention to nutrition and stunting reduction, later referred to as “Go-Baby-Go Plus” model). The latter model would encourage positive parent-child interactions that stimulate children's overall development, and in the specific areas of language, cognition, social-emotional, and motor skills. Outcomes of the integrated Go-Baby-Go-Plus (GBG/P) model were compared to outcomes of the SBC nutrition-focused (7–11) model alone through a quasi-experimental study lasting 14 months. The project was implemented in the Gavar and Vardenis regions of Gegharkunik province (administrative division) in the Republic of Armenia. The research questions of the overall study were:

Would the integration of an ECD counseling model (GBG Plus) within an integrated maternal, newborn, and child SBC delivery platform (7–11 model) lead to better child development outcomes?

Can early child development packages focusing on nurturing care (parental perception and behavior) support and improve child cognitive development in rural, low-income settings?

The hypothesis of this paper is that GBG/P is an effective, low-cost intervention, with an integrated approach that, due to its holistic characteristics and community structure implementation, produce improved physical growth and nourishment along with more optimal development outcomes (cognitive, language, physical) due to its alignment with relevant research in the area and its unique holistic integrated approach.

## 2 Materials and methods

### 2.1 Design

A non-randomized intervention-control study design was employed in two World Vision project areas in the Gegharkunik province. In total, 43 communities were included. Twenty-three communities with 680 study participants were allocated to the intervention arm (where the new integrated model GBG + 7–11 was implemented), while the remaining 20 communities with 630 participants served as the control arm (wherein the traditional 7–11 interventions focused on child nutrition were implemented). All children aged 0 to 24 months (born between February 1, 2013 and February 15, 2015) living in the 43 communities were recruited into the study and followed over a period of 14 months (February 2015–April 2016), during which the integrated GBG + 7–11 model was applied in the intervention sites, and 7–11 in the control sites.

### 2.2 Study tools and measurement

At the time of recruitment, a baseline study was conducted among 1280 children and their caretakers to ascertain that study arms were identical in terms of socio-demographic characteristics as well as child care practices (e.g., early learning support activities, father engagement in learning support, available toys, child books, disciplining practice), nutritional practices (e.g., dietary diversity, breastfeeding), and child nutrition status (stunting rate). At the start and endpoints, all parents were interviewed by trained data collectors using a standardized questionnaire that elicited information on demographics, socioeconomic status, child nutrition, and developmental promotion practices. Nurses assessed height and weight of children according to the standard World Health Organization (WHO) protocol, using advanced precision stadiometers (SECA 217, Germany) and electronic weighing scales (SECA 869, Germany).

Further, 270 randomly-selected children (140 at intervention sites and 130 at control sites) were assessed for neurocognitive development using the Bayley Scales of Infant and Toddler Development, third edition (BSID-III). The BSID-III is a diagnostic measure standardized and validated in the U.S., providing quotients for the following composites: cognition, language, and motor. The BSID-III was administered in children's homes by medical doctors trained in the use of the instrument by clinicians from Aga Khan University, Pakistan. The assessment of each child lasted about two hours, and evaluators were blinded to study arms. An appointment was scheduled by phone before the visit. Around 5% (*n* = 14) of the total children assessed using the BSID III were also scored by the supervisors.

### 2.3 Statistical analysis

First, the demographic and socioeconomic covariates of study participants' intervention and control arms were compared. Second, the association of child and family characteristics with each outcome of interest was tested by calculating the odds ratio (OR) for categories of variables and testing for statistical significance of deviation of OR from one. All covariates that were differently distributed across intervention and control arms—both at baseline and at the end of the study—as well as variables associated with the outcomes of interest were considered potential confounders for the intervention and outcomes of the interest relationship. A multivariate logistic regression model was used to assess adjusted effects of the intervention accounting for all confounding factors. STATA 12 software was used for all statistical analysis, and a *P* value <0.05 was considered significant.

### 2.4 Research ethics approval

Study protocol was approved by the institutional review boards of Yerevan State Medical University. All study participants provided their informed consent before enrollment.

### 2.5 Description of intervention

During the first phase of the intervention, 32 GBG facilitators were selected and participated in a three-day training of trainers (TOT) workshop. Following the post-test certification, the pairs of facilitators were provided with the facilitator guide, package of resources for the sessions (scissors, sewing thread, needle, glue, dolls, colorful textiles, ball, colorful markers, flip charts, pre-/post-tests, video films and equipment to display those), and educational materials for parents. Facilitators assigned groups to all parents of children aged 0–23 months and planned the subsequent sessions. The primary health care providers were involved in parent mobilization and ensured their participation. During the four-month period following the TOT, the GBG-trained facilitators conducted group sessions on positive parenting and the early stimulation technique for all mothers of children recruited into the study. In total, facilitators conducted 62 group sessions, each consisting of four meetings. Each parental group included 12–15 participants. Sessions included the provision of key messages and information, group discussions, role playing, watching video material, and preparing toys and books according to the curriculum. To ensure instructional fidelity, World Vision and partner staff conducted quality supervision using a monitoring form to assess the GBG facilitator; each pair was monitored at least once and given feedback for improvements. In addition, supportive supervision was introduced in primary health care facilities in selected communities to ensure that children enrolled in the study were screened for growth and development according to national protocols. Supportive supervision included on-the-job training of screening techniques, review of medical records, and counseling on observations during the health visit.

Based on reflection and learning, additional sessions tailored to needs and recommendations were developed and delivered following TOT during the second phase of implementation. In addition, 13 two-hour sessions were conducted for 179 grandmothers and 78 fathers. After mapping the eligible mothers who did not participate in the first GBG phase, an individualized approach was developed on how to reach out to each of those families through home visiting sessions.

## 3 Results

### 3.1 Characteristics of the participants and families per study arms

Characteristics of the 1265 children studied are summarized in Table 1; each study arm had similar age and sex structures. Children ranged, at end of the study, from 13 to 38 months of age, and 10% were born pre-term. The mean number of children per family was small (1.8), and most mothers (70%) had two children or fewer. Mothers were young (79% were under 30 years of age). Most parents (58% of mothers and 74% of fathers) had a secondary education, while the proportion of mothers and fathers who completed higher education was 18% and 12%, respectively. In 36% of households, fathers were absent for three or more months out of the past year due to seasonal work migration demands. Around half of the households (46%) included six to seven members, and 12% lacked access or consistent access to running water. Most household variables were similar in the two research arms. They differed, however, in twins (*P* = 0.000) and fuel used for cooking (*P* = 0.000). Those variables were controlled for in multiple variable analyses.

**Table 1:** Sociodemographic characteristics of surveyed population per study arms, April 2016, Armenia.

Characteristics	Control (*n* = 615)		Intervention (*n* = 650)		Total (*n* = 1265)		Chi^2^, *P* value
	Number	(%)	Number	(%)	Number	(%)	
Sex							
Male	318	(51.7)	350	(53.8)	668	(52.8)	chi^2^ [_df (1)_] = 0.446 Pr = 0.580
Female	297	(48.3)	300	(46.2)	597	(47.2)	
Age group							
13 to 25.9 month	291	(52.5)	323	(49.7)	614	(48.5)	chi2(2) = 0.714 Pr = 0.398
26 to 38 months	324	(53.2)	327	(50.3)	651	(51.5)	
Number of children at household under 18 yr.							
One child	132	(21.7)	144	(22.5)	276	(22.1)	chi2(5) = 9.52 Pr = 0.218
Two children	279	(45.8)	323	(50.5)	602	(48.2)	
Three children	129	(21.2)	110	(17.2)	239	(19.2)	
Four children	50	(8.2)	42	(6.6)	92	(7.4)	
Five children	11	(1.8)	17	(2.7)	28	(2.2)	
Six or more children	8	(1.3)	3	(0.5)	11	(0.9)	
Birth term - gestational age							
Full-term (37–40 weeks)	553	(90.8)	572	(89.5)	1125	(90.1)	chi2(1) = 0.58 Pr = 0.445
Pre-term (<37 weeks)	56	(9.2)	67	(10.5)	123	(9.9)	
Twins (two offsprings produced by the same pregnancy)							
Yes	19	(3.1)	3	(0.5)	22	(1.8)	**chi2**(**1**) = **12**.**6** **Pr** = **0**.**000**
No	590	(96.9)	636	(99.5)	1226	(98.2)	
Mother's education							
Eighth grade or below	18	(3.0)	18	(2.8)	36	(2.9)	chi2(4) =4.74 Pr = 0.192
Secondary	364	(59.8)	358	(56.0)	722	(57.9)	
Vocational and incomplete higher education	115	(18.9)	153	(23.9)	268	(21.5)	
Higher/college-university education	112	(18.4)	110	(17.2)	222	(17.8)	
Father's education							
Eighth grade or below	29	(4.8)	21	(3.3)	50	(4.0)	chi2(5) = 7.45 Pr = 0.059
Secondary	466	(76.5)	461	(72.1)	927	(74.3)	
Vocational and incomplete higher education	53	(8.7)	71	(11.1)	124	(9.9)	
Higher education	61	(10.0)	86	(13.5)	147	(11.8)	
Maternal age in years							
<24 years	203	(33.3)	247	(38.7)	450	(36.1)	chi2(5) = 3.81 Pr = 0.149
25–30 years	273	(44.8)	261	(40.8)	534	(42.8)	
30 and over	132	(21.7)	131	(20.5)	263	(21.1)	
Fathers not present at household over 3 months in the past 12 months							
Yes	211	(34.6)	219	(34.3)	430	(34.5)	chi2(1) =0.035 Pr = 0.852
No	391	(64.2)	415	(64.9)	806	(64.6)	
Household size (number of people living together in one house)							
3–5 people	180	(29.6)	176	(27.5)	356	(28.5)	chi2(2) = 2.62 Pr = 0.270
6–7 people	264	(43.3)	306	(47.9)	570	(45.7)	
8 and above	165	(27.1)	157	(24.6)	322	(25.8)	
Access to water							
Accessible	513	(84.2)	543	(85.0)	1056	(84.6)	chi2(3) =1.62 Pr = 0.656
Somewhat accessible	29	(4.8)	37	(5.8)	66	(5.3)	
Very difficult to access	45	(7.4)	40	(6.3)	85	(6.8)	
Not accessible	22	(3.6)	19	(3.0)	41	(3.3)	
Fuel used for cooking in the house							
Solid fuel (coal, wood, animal dunk)	94	(15.4)	57	(8.9)	151	(12.1)	**chi2**(**1**) = **12**.**4** **Pr** = **0**.**000**
Electricity or gas	515	(84.6)	581	(90.9)	1096	(87.8)	
Challenge to feed the family during last month							
Yes	165	(27.1)	166	(26.0)	331	(26.5)	chi2(1) = 0.20 Pr = 0.655
No	444	(72.9)	473	(74.0)	917	(73.5)	
Wealth score							
Very low	130	(21.3)	121	(18.9)	251	(20.1)	chi2(4) = 7.64 Pr = 0.106
Low	133	(21.8)	114	(17.8)	247	(19.8)	
Average	115	(18.9)	133	(20.8)	248	(19.9)	
High	121	(19.9)	128	(20.0)	249	(20.0)	
Very high	106	(17.4)	142	(22.2)	248	(19.9)	
Geographical Region							
Gavar	301	(49.4)	336	(52.6)	637	(50.4)	chi2(1) = 0.96 Pr = 0.328
Vardenis	314	(51.6)	314	(49.1)	628	(49.6)	

### 3.2 Effects of intervention

#### 3.2.1 Effects of intervention on child care and nutrition practices

While child care and practices were similar at baseline, the odds of receiving minimum dietary diversity at the intervention site were 55% higher compared to control than at endline, which was statistically significant after controlling for all possible confounding factors (aOR = 1.55, 95%CI 1.10–2.19, *P* = 0.013). Likewise, parents in the intervention communities demonstrated better support for learning and disciplining practices compared to controls (aOR = 2.22, 95%CI 1.19–4.16, *P* = 0.012). Violating disciplining practice was comparable across the study arm, indicating no evidence of the effectiveness of interventions on these practices (Table 2).

**Table 2:** Effect of intervention on caregiver's child care and nutrition practice.

Care and nutrition practice	Control (*n* = 615)		Intervention (*n* = 650)		Uni-variate analysis			Multivariate analysis^a^		
	N	(%)	N	(%)	OR	(95% CI)	*P* value	aOR	(95% CI)	*P* value
Minimum dietary diversity										
Under 4 food groups	91	(15.0)	63	(9.9)	Ref			Ref		
4 food groups and above	515	(85.0)	573	(90.1)	1.61	(1.14–2.27)	0.006	1.55	(1.10–2.19)	**0**.**013**
Support for learning										
Under 4 activities	39	(6.3)	26	(4.0)	Ref			Ref		
4 or more activities	576	(93.7)	624	(96.0)	1.63	(0.98–2.71)	0.059	2.22	(1.19–4.16)	**0**.**012**
Violating disciplining										
No	109	(17.7)	119	(18.3)	Ref					
Yes	506	(82.3)	531	(81.7)	0.96	(0.72–1.28)	0.787			

a Adjusted for co-variate “solid fuel used for cooking.”

#### 3.2.2 Effect of intervention on Bayley total composite and cognitive, motor and language subscale composites

Continuous variables for each of the composite cognitive, language and motor subscales were split using −1 standard deviations (SD) (<85) to create two categories to determine groups with normal development and with developmental delays. An aggregated composite variable was created to examine the overall development outcomes: children who achieved at least a score of 85 in cognitive, language, and motor composite scores were categorized into “higher total composite” groups, and those who scored below 85 in any of composite subscales were categorized in the “lower total composite” group. Overall, the OR of association of the intervention and the outcome of interest adjusted for all confounding factors showed a moderately statistically significant relationship with Bayley Total Composite: 71.4% of children in the intervention arm versus 59.2% in the control arm yielded over 85 in all three composite subscales. This relationship became even stronger after accounting for the effect of possible confounding factors in the multivariate logistic regression model, showing that children in the intervention arm had 83% higher odds of total composite score compared to children in the control arm (aOR = 1.83, 95% CI 1.08–3.09, *P* value = 0.025). Although children in intervention communities scored higher than the controls in all composite subscales, this difference was not statistically significant (Table 3).

**Table 3:** Effect of intervention on child developmental outcomes.

Developmental outcomes	Control (*n* = 130)		Intervention (*n* = 140)		Univariate analysis			Multivariate analysis		
	N	(%)	N	(%)	OR	(95% CI)	*P* value	aOR	(95% CI)	*P* value
Total composite^a^										
Lower	53	(40.8)	40	(28.6)	Ref			**Ref**		
Higher (at least 85 in all 3)	77	(59.2)	100	(71.4)	1.72	(1.04–2.86)	**0**.**036**	**1**.**83**	(**1**.**08**–**3**.**09**)	**0**.**025**
Cognitive composite^b^										
Below 85	32	(24.6)	25	(17.9)	Ref			Ref		
85 and above	98	(75.4)	115	(82.1)	1.50	(0.83–2.71)	0.175	1.50	(0.83–2.71)	0.175
Language composite^c^										
Below 85	22	(16.9)	18	(12.9)	Ref			**Ref**		
85 and above	108	(83.1)	122	(87.1)	1.38	(0.70–2.71)	0.349	1.68	(0.83–3.43)	0.151
Motor composite^d^										
Below 85	32	(24.6)	27	(19.3)	Ref			Ref		
85 and above	98	(75.4)	113	(80.7)	1.36	(0.77–2.44)	0.291	1.36	(0.77–2.44)	0.291

a Total composite: adjusted for wealth score.

b Cognitive composite: no covariate to distort OR over 10%.

c Language composite: adjusted for fuel, age and wealth score.

d Motor composite: no covariate to distort OR over 10%.

#### 3.2.3 Effect of intervention on nutrition status

There was strong evidence that child nutrition practices improved in intervention sites, mainly indicated by a statistically significant increase in food diversity in the intervention site versus controls, as shown in Table 1. This improvement in practice was not translated into nutrition status of children, however: prevalence of stunting at the intervention and control sites were almost equal (aOR =1.11, 95% CI 0.83–1.48, *P* value = 0.501) (Table 4).

**Table 4:** Effect of intervention on Bayley total and cognitive, motor and language composite subscales.

Nutrition status	Control (*n* = 615)		Intervention (*n* = 650)		Univariate analysis			Multivariate analysis		
	N	(%)	N	(%)	OR	(95% CI)	*P* value	aOR	(95% CI)	*P* value
Not stunted	487	(81.3)	519	(80.7)	Ref			**Ref**		
Stunted	112	(18.7)	124	(19.3)	1.04	(0.78–1.38)	**0**.**792**	**1**.**11**	(**0**.**83**–**1**.**48**)	**0**.**501**

Adjusted for preterm birth, fuel, and sex.

Since the populations in the two selected regions differed notably in sociodemographic characteristics, repeated analysis stratified by region assessed geographic variability. While analyzing the effect of interventions on children's developmental outcomes stratified by region, it appeared that in the Vardenis region the effect of the intervention on the total composite score was even more pronounced compared to overall effect (OR = 3.38 vs. 1.72). In addition, by their language and motor developmental outcomes, children in intervention communities in the Vardenis region yielded much higher scores compared to control communities, even adjusted for all confounding factors (Table 5).

**Table 5:** Adjusted effect of intervention on Bayley total and cognitive, motor and language composite sub-scales in Vardenis region only.

Developmental outcomes	aOR of intervention vs. control	95% CI	*P*-value
Total composite subscale^a^	3.38	(1.53–7.61)	0.003
Cognitive composite subscale^b^	1.95	(0.85–4.49)	0.115
Language composite subscale^c^	5.32	(1.36–20.86)	0.016
Motor composite subscale^d^	3.04	(1.26–7.33)	0.013

a Adjusted for fuel, maternal education, and wealth score.

b Adjusted for maternal education.

c Adjusted for fuel, child age, paternal education.

d Adjusted for fuel and maternal education.

## 4 Discussion

This research began with the assumption that by merging an ECD intervention focused on parental education to promote nurturing care into an established, community-based MCH delivery platform, child development outcomes would improve. Parenting programs are operationally defined as interventions or services aimed at improving parenting interactions, behaviors, knowledge, beliefs, attitudes and practices (Britto et al., 2017). Researchers prospectively collected measurements of parental behavior and child development outcomes (cognitive, language, and motor composites) of a cohort of children 0–24 months of age and their caretakers for 14 months.

Overall, children exposed to World Vision's integrated ECD intervention (GBG plus 7–11 nutrition-focused model) had significantly better total composite score on the BSID scale than their counterparts who experienced only 7–11 nutrition-focused interventions. Effects were particularly strong for children living in socioeconomically depressed geographical areas (Vardenis region).

The difference was not significant, however, between the two arms in terms of nutritional status (stunting prevalence). The parenting component of the intervention focused on nurturing care showed significant differences between intervention and control groups in nutrition-related parental practices (diet diversity) and stimulation (support for learning). Conversely, the intervention did not show significant differences on the safety-related component (violating discipline). Several recent systematic studies on parenting support have demonstrated deep benefits on childhood development (Aboud and Yousafzai, 2015; Rao et al., 2014; Britto et al., 2015; and Britto et al., 2017), especially in cognitive and language development. Likewise, these same studies did not find any significant impact on child growth (Britto et al., 2017).

The delivery platform used by World Vision's model—group sessions—showed similar outcomes to program models using similar delivery platform, such as Pastoral del Niño in Paraguay (Peairson et al., 2008), higher outcomes than program models using home visits only, such as Roving Caregivers in Jamaica (Powell, 2004), and programs that used both group sessions and home visits, such as the Brazilian model (Eickmann et al., 2003).

The overall results of the integrated World Vision model seem to be aligned with previously published results, validating its overall population target, service package and delivery platform in terms of both quality and effectiveness of ECD services and intended outcomes. Integrated-GBG and other research are aligned with the core evidenced-based belief that the most effective ECD programs are those that combine infant stimulation or parenting education with nutrition. These focuses have additive effects when joined together, especially when they are high intensity, targeted toward more disadvantaged children, and are of long duration.^1^ In this manner, integrated GBG is an effective, holistic, low-cost intervention ($1 per child) that can be executed by health care workers during home or clinic visits. Given further community-based training, it is probable that even less expensive approaches could be deployed, such as local parent-to-parent support groups.

Given the absence of randomization in the study design, potential imbalance between the observed and counterfactual states were addressed by controlling for confounding variables with regression techniques, with its inherent limitations on validity and interpretation of results. Short duration of intervention application might have influenced the absence of nutritional impact in the intervention arm. Study limitations are mostly related to short duration of project (14 months of implementation) study statistical power and to the use of an overall quasi-experimental research design. Larger samples that were randomly selected would have provided a more valid estimation of measurement.

## 5 Conclusion

The expansion of early childhood development programs is a fundamental step to meeting the sustainable development goals that aspire to reach the >200 million children under 5 in the developing world who currently do not fulfill their potential for development. Go Baby Go Plus in Armenia has proven to be an effective, cost-efficient program that helps to ensure children reach their full potential in an essential time of their life. Integrated Go Baby Go research aligns with other relevant research in the area. There is still room for improvement, however, as the research recognizes when it calls for further development of the curriculum based on accumulated experiences, for instance, that “given further training, even less expensive approaches could be deployed, such as local parent-to-parent support groups.”

## Authors' contributions

Conception and design of the research (Alfonso Rosales, Viktoria Sargsyan), acquisition of data (Karine Abelyan, Arax Hovhannesyan, Kristine Ter-Abrahanyan), analysis and interpretation of data (Arax Hovhannesyan, Alfonso Rosales), obtaining of funding (Dennis Cherian, Alfonso Rosales, and Kimberly Quanstrum Jillson), writing of manuscript (Alfonso Rosales and Viktoria Sargsyan), critical revision of manuscript (Dennis Cherian).

## Funding

This study and publication was funded by The Bill & Melinda Gates Foundation (BMFG 03360000013) as part of The Grand Challenge Exploration Round 13 (OPP 1119453).

## Ethics approval and consent to participate

Study protocol was approved by the institutional review boards of Yerevan State Medical University. All study participants provided their informed consent before enrollment.

## Competing interests

The authors declare that they have no competing interests.

## Footnotes

1 Launch of the 2016 *Lancet* Series “Advancing Early Childhood Development: from Science to Scale” in Washington, DC, USA, on Oct 5, 2016.
